# Global incidence and prevalence of idiopathic pulmonary fibrosis

**DOI:** 10.1186/s12931-021-01791-z

**Published:** 2021-07-07

**Authors:** Toby M. Maher, Elisabeth Bendstrup, Louis Dron, Jonathan Langley, Gerald Smith, Javaria Mona Khalid, Haridarshan Patel, Michael Kreuter

**Affiliations:** 1 Keck School of Medicine, The University of Southern California, Los Angeles, CA USA; 2 National Heart and Lung Institute, Imperial College, London, UK; 3 Center for Rare Lung Diseases, Department of Respiratory Diseases and Allergy, Aarhus University Hospital, Aarhus, Denmark; 4 Cytel, Vancouver, BC Canada; 5 Global Medical Affairs, Galapagos, NV, Mechelen, Belgium; 6 Evidence Generation and Epidemiology, Global Medical Affairs, Galapagos NV, Mechelen, Belgium; 7 Center for Interstitial and Rare Lung Diseases, Department of Pneumology and Respiratory Critical Care Medicine, University of Heidelberg, Heidelberg, Germany; 8 German Center for Lung Research, Heidelberg, Germany

**Keywords:** Case definition, Epidemiology, Idiopathic pulmonary fibrosis, Interstitial lung disease, Modeling, Rare disease

## Abstract

**Background:**

Idiopathic pulmonary fibrosis (IPF) is a progressive debilitating lung disease with considerable morbidity. Heterogeneity in epidemiologic studies means the full impact of the disease is unclear.

**Methods:**

A targeted literature search for population-based, observational studies reporting incidence and/or prevalence of IPF from January 2009 to April 2020 was conducted. Identified studies were aggregated by country. For countries with multiple publications, a weighted average was determined. Incidence and prevalence data were adjusted for between-study differences where possible. The final model included adjusted estimates of incidence and prevalence per 10,000 of the population with 95% confidence intervals. As prevalence estimates vary depending on the definitions used, estimates were based on a specific case definition of IPF.

**Results:**

Overall, 22 studies covering 12 countries met the inclusion criteria, with 15 reporting incidence and 18 reporting prevalence estimates. The adjusted incidence estimates (per 10,000 of the population) ranged from 0.35 to 1.30 in Asia–Pacific countries, 0.09 to 0.49 in Europe, and 0.75 to 0.93 in North America. Unadjusted and adjusted incidence estimates were consistent. The adjusted prevalence estimates ranged from 0.57 to 4.51 in Asia–Pacific countries, 0.33 to 2.51 in Europe, and 2.40 to 2.98 in North America. South Korea had the highest incidence and prevalence estimates. When prevalence estimates were compared to country-specific rare disease thresholds, IPF met the definition of a rare disease in all countries except South Korea. There were notable geographic gaps for IPF epidemiologic data.

**Conclusions:**

Due to differences in study methodologies, there is worldwide variability in the reported incidence and prevalence of IPF. Based on the countries included in our analysis, we estimated the adjusted incidence and prevalence of IPF to be in the range of 0.09–1.30 and 0.33–4.51 per 10,000 persons, respectively. According to these prevalence estimates, IPF remains a rare disease. For consistency, future epidemiologic studies of IPF should take age, sex, smoking status, and the specificity of case definitions into consideration.

**Supplementary Information:**

The online version contains supplementary material available at 10.1186/s12931-021-01791-z.

## Background

Idiopathic pulmonary fibrosis (IPF) is a rare chronic progressive disease of unknown etiology that affects both physical and emotional well-being [1–3]. It is characterized by irreversible loss of lung function due to fibrosis, which manifests as symptoms of increasing cough and dyspnea and impaired quality of life [2–6]. Lung transplantation is limited to a minority of patients and patients primarily rely on antifibrotic therapy plus several supportive/palliative treatments. Despite recent advances, current IPF therapies only slow disease progression and prognosis is poor, with a median survival of 2–3 years if left untreated [7]. Accordingly, reliance on healthcare services is considerable, contributing to a marked socioeconomic burden of disease [8, 9].

Epidemiology estimates of IPF are derived using various data sources. For those using claims databases, it is important to differentiate between specific versus non-specific case definitions of IPF, as estimates can vary drastically depending on the definitions used [10–13]. A specific case definition is obtained from an accurate diagnosis of IPF, which requires observation of clinical characteristics as well as confirmation of specific pulmonary patterns via high-resolution chest imaging and sometimes lung biopsy [1]. However, some patients are diagnosed with IPF without precise diagnostic procedures and as such can only be considered under a broad (non-specific) case definition.

Single studies describing the epidemiology of IPF can also be misleading if age, sex, and other risk factors are not taken into consideration [1, 10]. The mean age of IPF patients is around 65–70 years, with incidence increasing with age [14–16]. Globally, patient numbers are rising, which may be attributed to, among other causes, an aging population, a higher degree of disease awareness and improved diagnostic tools [17–19]. Furthermore, IPF affects males more than females [10], and risk factors such as smoking [20, 21], metal/wood dust inhalation [22], and genetic factors [23, 24] are frequently recorded as being associated with development of IPF.

Overall, owing to diagnostic challenges, updated diagnostic criteria, and differences in study methodologies there is substantial heterogeneity between studies providing estimated epidemiology data in IPF [1, 10], impacting the understanding of global disease burden. Indeed, a detailed knowledge of the incidence and prevalence of IPF provides additional disease understanding that is crucial for therapeutic and healthcare system planning, particularly when considering the socioeconomic burden of the disease. By re-evaluating the published literature, this study sought to produce adjusted incidence and prevalence for IPF by country.

## Methods

This was a targeted literature review to identify studies estimating epidemiologic measures of IPF published between 2009 and 2020. Statistical modeling was applied to the epidemiologic estimates obtained from the identified studies to provide adjusted incidence and prevalence data.

### Study design and data processing

The PubMed and EMBASE databases were searched for population based, observational studies from January 2009 to January 2019 using a search strategy derived from the following PICO (population, intervention, comparison, outcome) formulation: (i) patients with IPF (no restriction on case definitions); (ii) any intervention; (iii) any comparator; (iv) with outcomes including quantitative measures of IPF incidence (authors’ definition) and IPF prevalence (authors’ definition) (Additional file 1: Table S1). EMBASE was also searched to identify congress abstracts from 2014 to 2019, and supplementary gray literature searches were performed. We conducted a secondary supplemental search utilizing the same search terms between January 2019 and April 2020. No publications which met the threshold for inclusion in our analysis were identified through this supplementary search. Identified studies were aggregated at country-level and estimates further categorized based on the case definition (“specific” [i.e. narrow] or “broad”) used to identify patients with IPF. Studies were classified by two individuals in a blinded manner with adjudication by a third person where opinions differed with regards to the classification of the IPF identification. Collectively, studies utilizing broad classification criteria tended toward a generalized search of pertinent medical records for diagnostic classification according to the International Classification of Diseases (ICD) or a related coding system, without any additional diagnostic steps being undertaken. Studies reporting specific classifications typically required confirmatory imaging and/or pathology in addition to the ICD code classification or required review by medically trained staff.

### Statistical analysis

Incidence and prevalence data were adjusted to fit a negative binomial general linear model developed under a fixed-effects framework, using a study population offset parameter to adjust for population size of each study. An initial “full model” included age, sex, study year, diagnostic criteria, study region/country, and population size; any covariates in the model that were not significant at an alpha-level of 0.05 were removed (except age and sex, which were included in all models). In instances where data on age or proportion of male patients were not directly provided, appropriate estimates for a given study population were used or a value was imputed using the average of all the other studies. The outcome variable in the model was the total number of IPF cases, whether for incidence or prevalence. For countries with multiple publications, a weighted average was determined using the underlying study population number as the weighting coefficient. The final model included adjusted estimates of incidence and prevalence per 10,000 of the population with 95% confidence intervals. Model-associated adjustments for prevalence estimates are provided in Additional file 1: Table S2. For prevalence estimates, a sensitivity analysis was performed using broad IPF case definitions.

Prevalence estimates were compared to country-specific rare disease thresholds [25–31]. For countries where a threshold of cases, as opposed to a prevalence, is utilized, the prevalence estimates were multiplied by the countries 2020 United Nations population estimate [32] to determine a total number of estimated cases.

## Results

### Study selection

**Fig. 1:**
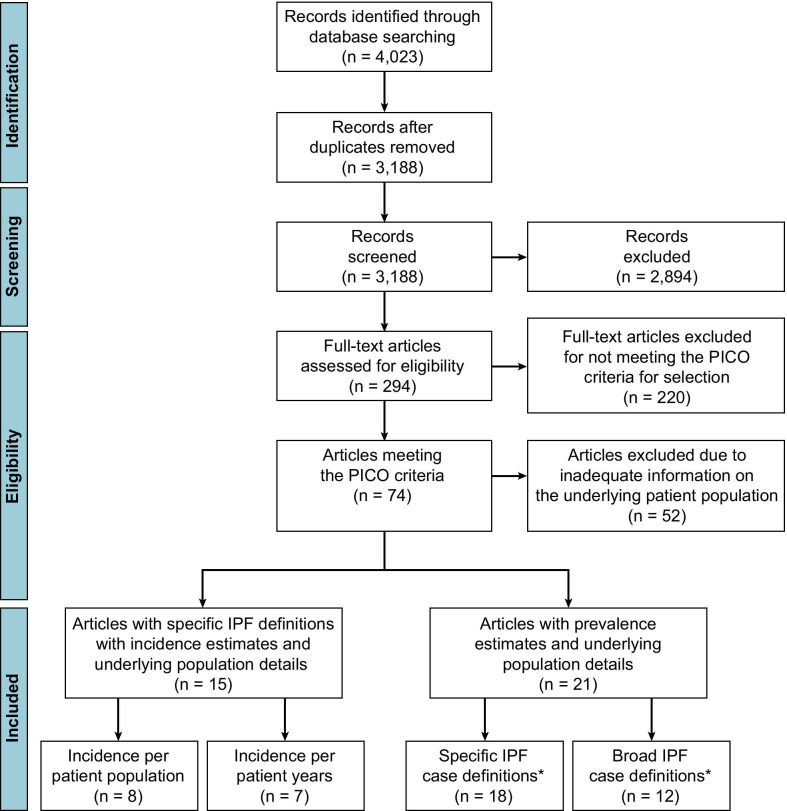
An overview of articles reviewed and study flow. *Some studies included both a broad and a specific case definition. *IPF* idiopathic pulmonary fibrosis, *PICO* population, intervention, comparison, outcome

Following the removal of duplicate articles, the literature search yielded 3188 hits (Fig. 1). The abstracts of these publications were reviewed and the full-text versions of 294 manuscripts were examined against the PICO criteria for selection (Additional file 1: Table S1). Of the 74 articles that met the criteria, 22 provided incidence and/or prevalence, IPF case identification descriptions, and details on the underlying patient populations. The included studies were classified as “specific” or “broad” according to how IPF patients were identified [11–13, 33–50] (Additional file 1: Table S3). Of those studies reporting a specific IPF case definition, 15 reported incidence estimates and underlying population details [11–13, 33, 35, 37, 38, 40, 41, 43, 44, 46, 47, 51–53] (Additional file 1: Table S4) and 18 described prevalence estimates and underlying population details [11–13, 33, 35, 37–47, 50, 52] (Additional file 1: Table S5). Of the 15 studies reporting incidence estimates, five used primary databases including medical charts and other direct sources and 10 used secondary research databases including claims data. In addition, eight studies reported incidence per population and seven reported incidence per patient-year. For prevalence estimates, eight studies used primary databases and 10 used secondary databases. In total, the studies covered 12 countries and corresponded to review of 229,611,497 patient records globally.

### Estimated incidence

**Fig. 2:**
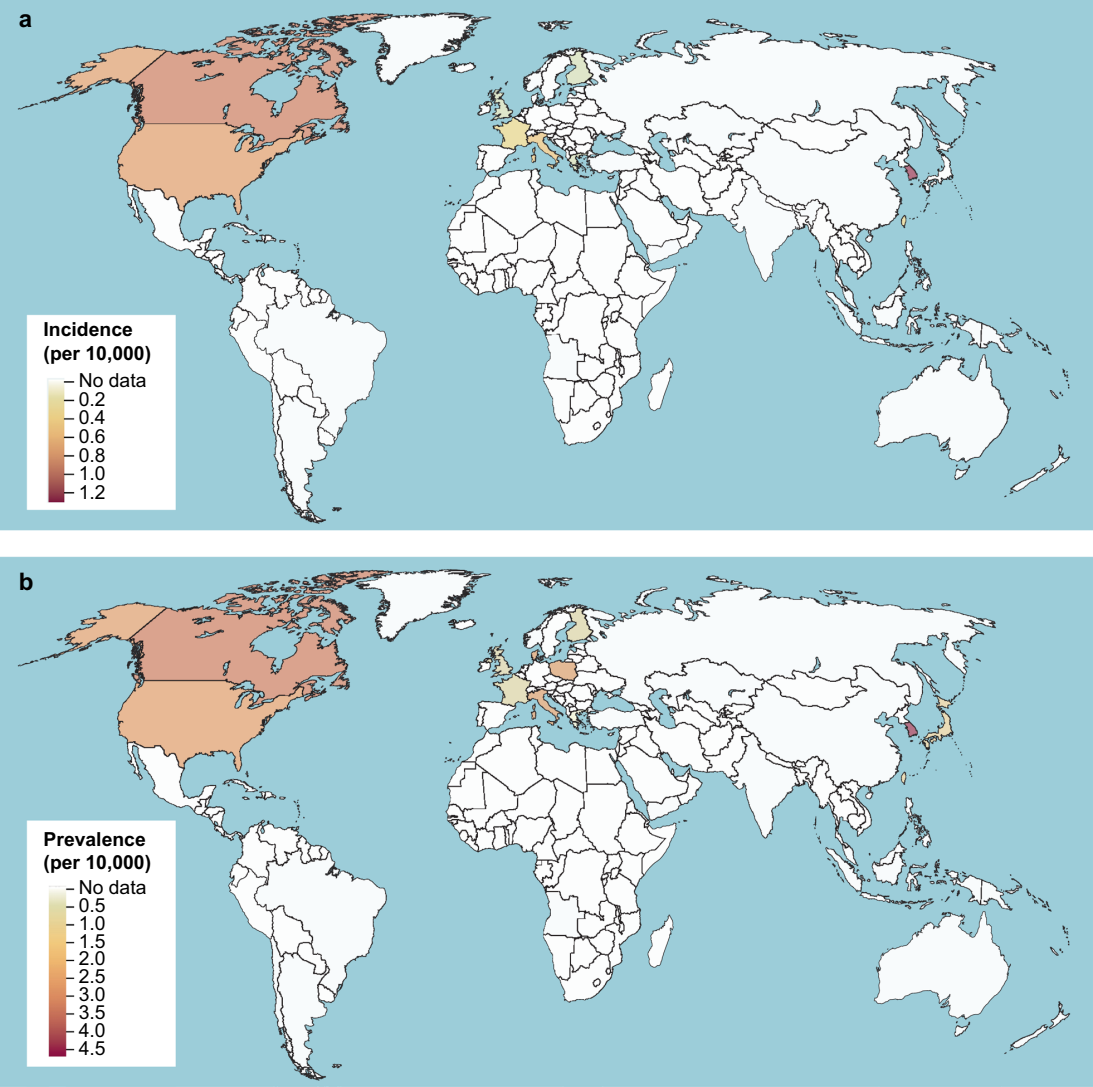
Global heat maps of adjusted IPF incidence (**a**) and prevalence (**b**) for included studies with specific IPF definitions. *IPF* idiopathic pulmonary fibrosis

The adjusted incidence estimates (per 10,000 of the population) for each country ranged from 0.35 to 1.30 in Asia–Pacific countries, 0.09 to 0.49 in Europe, and 0.75 to 0.93 in North America (Table 1). Overall, unadjusted and adjusted incidence estimates were similar. Both age and country were identified as statistically significant variables within the model. There are clear epidemiologic knowledge gaps in substantial geographic regions including Africa, South America, South Asia, and the Middle East (Fig. 2a).

**Table 1:** IPF incidence estimates per country

Country	Study	Publication year	Mean unadjusted incidence (per 10,000)	Adjusted incidence (per 10,000)	95% CI adjusted incidence (per 10,000)
Asia–Pacific					
South Korea	Han et al. [51]	2013	0.19	1.30	(0.62, 2.74)
	Kim et al. [41]	2017	1.31		
	Lee et al. [44]	2016	1.29		
	Combined		1.28		
Taiwan	Lai et al. [43]	2012	0.31	0.35	(0.17, 0.72)
Europe					
Finland	Kaunisto et al. [40]	2015	0.13	0.10	(0.04, 0.22)
France	Duchemann et al. [35]	2017	0.28	0.31	(0.07, 1.29)
Greece	Karakatsani et al. [52]	2009	0.09	0.09	(0.04, 0.18)
Italy	Agabiti et al. [33]	2014	0.93	0.49	(0.27, 0.91)
	Harari et al. [37]	2016	0.26		
	Combined		0.48		
United Kingdom	Strongman et al. [13]	2018	0.12	0.14	(0.06, 0.32)
North America					
Canada	Hopkins et al. [38]	2016	0.90	0.93	(0.54, 1.60)
	Tarride et al. [12]	2018	2.17		
	Combined		1.11		
United States	Fernández Pérez et al. [11]	2010	0.88	0.75	(0.28, 2.00)
	Raghu et al. [47]	2014	2.42		
	Raghu et al. [46]	2016	0.26		
	Combined		0.64		

*CI* confidence interval, *IPF* idiopathic pulmonary fibrosis

### Estimated prevalence

The adjusted prevalence estimates (per 10,000 of the population) for each country ranged from 0.57 to 4.51 in Asia–Pacific countries, 0.33 to 2.51 in Europe, and 2.40 to 2.98 in North America (Table 2). Overall, unadjusted and adjusted prevalence estimates were similar. Notable exceptions were South Korea and the United States, both of which demonstrated higher adjusted prevalence when compared to unadjusted estimates (4.51 vs. 3.70 and 2.40 vs. 1.37, respectively). Conversely, the United Kingdom demonstrated a modestly reduced adjusted versus unadjusted IPF prevalence (0.78 vs. 1.16). The adjusted prevalence estimates (per 10,000 of the population) from the sensitivity analysis (using broad IPF case definitions) for each country ranged from 0.79 to 5.67 (Table 3).

**Table 2:** IPF prevalence estimates per country

Country	Study	Publication year	Mean unadjusted prevalence (per 10,000)	Adjusted prevalence (per 10,000)	95% CI Adjusted prevalence (per 10,000)	Rare disease threshold (per 10,000)	Rare disease threshold met?
Asia–Pacific							
Japan	Kondoh et al. [42]	2016	0.59	0.89	(0.51, 1.55)	< 50,000 cases^a^ [25]	NA
	Natsuizaka et al. [45]	2014	1.00				
	Combined		0.79				
South Korea	Kim et al. [41]	2017	3.52	4.51	(2.99, 6.79)	< 20,000 cases^a^ [26]	NA
	Lee et al. [44]	2016	3.89				
	Combined		3.70				
Taiwan	Lai et al. [43]	2012	0.49	0.57	(0.34, 0.94)	1 [27]	Yes
Europe							
Denmark	Hyldgaard et al. [39]	2014	1.01	1.17	(0.56, 2.44)	1–2 [28]	Yes (upper CI out of bounds)
Finland	Kaunisto et al. [40]	2015	0.86	0.65	(0.36, 1.18)	5 [29]	Yes
France	Duchemann et al. [35]	2017	0.82	0.94	(0.44, 1.99)	5 [29]	Yes
Greece	Karakatsani et al. [52]	2009	0.34	0.33	(0.21, 0.53)	5 [29]	Yes
Italy	Agabiti et al. [33]	2014	2.56	2.37	(1.38, 4.09)	5 [29]	Yes
	Harari et al. [37]	2016	2.12				
	Combined		2.46				
Poland	Szafrański [50]	2012	2.56	2.51	(1.55, 4.05)	5 [29]	Yes
United Kingdom	Strongman et al. [13]	2018	1.16	0.78	(0.38, 1.63)	5 [29]	Yes
North America							
Canada	Hopkins et al. [38]	2016	2.00	2.98	(1.7, 5.19)	5 [30]	Yes (upper CI out of bounds)
	Tarride et al. [12]	2018	7.27				
	Combined		2.98				
United States	Fernández Pérez et al. [11]	2010	2.81	2.4	(1.33, 4.34)	< 200,000^a^ [31]	Yes
	Raghu et al. [47]	2014	11.1				
	Raghu et al. [46]	2016	0.67				
	Combined		1.37				

*CI* confidence interval, *IPF* idiopathic pulmonary fibrosis, *NA* not applicable

^a^Number of cases provided rather than threshold

**Table 3:** IPF prevalence estimates utilizing broad case definitions per country

Country	Study	Publication year	Mean unadjusted prevalence (per 10,000)	Adjusted prevalence (per 10,000)	95% CI Adjusted prevalence (per 10,000)	Rare disease threshold (per 10,000)	Rare disease threshold met?
Asia–Pacific							
Taiwan	Lai et al. [43]	2012	0.64	0.79	(0.36, 1.74)	1 [27]	Yes (upper CI out of bounds)
Europe							
Italy	Agabiti et al. [33]	2014	2.56	3.08	(1.11, 8.55)	5 [29]	Yes (upper CI out of bounds)
	Harari et al. [37]	2016	3.61				
	Combined		3.27				
United Kingdom	Snell et al. [49]	2016	5.00	4.06	(1.98, 8.33)	5 [29]	Yes (upper CI out of bounds)
	Strongman et al. [13]	2018	3.88				
	Combined		4.16				
North America							
Canada	Hopkins et al. [38]	2016	4.18	3.99	(1.73, 9.23)	5 [30]	Yes (upper CI out of bounds)
	Tarride et al. [12]	2018	7.84				
	Combined		4.86				
United States	Esposito et al. [36]	2015	12.52	5.67	(1.74, 18.47)	< 200,000^a^ [31]	Yes (upper CI out of bounds)
	Fernández Pérez et al. [11]	2010	6.30				
	Raghu et al. [47]	2014	18.1				
	Raghu et al. [46]	2016	0.49				
	Raimundo et al. [48]	2016	1.98				
	Combined		2.96				

*CI* confidence interval, *IPF* idiopathic pulmonary fibrosis

^a^Number of cases provided rather than threshold

South Korea was the only country where the threshold for rare disease status (< 20,000 cases [26]) was exceeded by the adjusted prevalence estimate (4.51/10,000, equating to approximately 23,136 patients [assuming a population of 51.3 million] [32]), although the unadjusted estimate was within the rare disease criteria (3.70/10,000, equating to approximately 18,981 patients) (Table 2). Within the sensitivity analysis using the broader definitions of IPF, IPF prevalence estimates still met rare disease thresholds although the upper confidence interval exceeded the threshold in all cases (Table 3).

Both age and country were identified as statistically significant variables within the model. Each year increase in average age was associated with a 6.2% increase in IPF prevalence over the unadjusted estimate. Geographic evidence gaps for prevalence were similar to those observed for incidence (Fig. 2b).

## Discussion

To our knowledge, this is the first targeted literature review including a model for adjusted analyses of IPF incidence and prevalence. Of the countries analyzed, estimates of the adjusted incidence of IPF are in the range of 0.09 to 1.30 per 10,000 persons globally. Overall, the countries with the highest incidence of IPF are South Korea, Canada, and the United States. Fewer countries were available to evaluate when compared with the prevalence model.

Based on the countries included in our analysis, estimates of the adjusted prevalence of IPF are in the range of 0.33 to 4.51 per 10,000 persons globally. Because most studies had similar proportions of male patients and age distributions, the IPF estimates remained relatively unchanged between unadjusted and adjusted prevalence. Overall, the countries with the highest apparent prevalence of IPF include South Korea, Canada, Poland, the United States, and Italy, although the extent to which variations reflect true differences in prevalence rather than methodologic differences is open to question.

In all but one country (South Korea), IPF would be classified as a rare disease according to national guidelines. South Korea utilizes very stringent criteria for defining rare disease status of < 20,000 cases (an estimated prevalence of < 3.91/10,000 persons based on a population of 51.3 million). This is somewhat lower than the 5/10,000 threshold used by most European countries. Regardless, for South Korea, the mean adjusted prevalence is the highest of the countries evaluated and around a third greater than the country with the next highest adjusted prevalence (Canada). This difference may be due to an overestimation of cases due to the study populations (elderly with a high proportion of male patients), the definitions used in the South Korean studies, or due to genetic or environmental factors. For example, in 2011, an increase in lung injuries was observed in South Korea due to humidifier disinfectant use [54]. South Korea has also experienced high levels of particulate matter air pollution [55], which might be associated with the incidence of IPF [56].

Broadly, trends were consistent between the incidence and prevalence models. However, compared to other countries Taiwan ranked differently for incidence and prevalence. Taiwan had the fifth highest incidence of IPF (out of nine countries), yet in the prevalence model it was the second lowest behind Greece (out of 12 countries). The reason for this is unclear, as in both cases Taiwan was only subject to mild alterations in point-estimates for incidence and prevalence. The large Taiwan study showed evidence of a continual shift to greater IPF burden across the study period (1997–2007), and it is conceivable that there is simply a lag between the increased incidence observed and the associated prevalence [43]. However, the study also indicated that the median time from diagnosis to death was 0.7 years based on specific IPF case definitions, compared with 3.47 years in a comparable study from the United States [11, 43]. The shorter survival time recorded in Taiwan, which may have been partly due to delayed diagnosis of IPF and less access to specific IPF treatments at the time of the study [43, 57], could account for the lower observed prevalence.

Overall, the primary prevalence analyses were comparable with the sensitivity analyses. When the broader IPF definition was used to identify patients, the estimates of IPF prevalence increased compared with the specific definition. The broader definition can result in a considerably larger number of patients falsely being classified as having IPF. In the study from the United States by Raghu et al., the broad case subgroup enrolled approximately 60% more patients than the specific case subgroup [46]. Indeed, Strongman and colleagues noted a nearly threefold difference in IPF prevalence in the UK when utilizing a broad versus a specific IPF case definition [13]. In our study, when utilizing broad case definitions, the inference is similar to the principal findings, that there is substantial between-country heterogeneity.

This study has some limitations. A relatively small number of studies are included with high heterogeneity between them including differences in case definitions, type of database analyzed, and timing of data collection. For example, data were collected earlier for some countries (such as Greece [52]) and may provide an underestimate of incidence and prevalence as diagnostic criteria, assessments and use of a multidisciplinary team approach to diagnosis and care have evolved over time [58]. However, the coding for IPF has not altered in line with changes to the guidelines. As such, we do not anticipate that changes in the way we diagnose IPF have had a major impact on incidence and prevalence data. Of note, any potential impact of changes in diagnostic approach on IPF epidemiology are likely compounded by reported increases in the incidence of IPF over time [59]. Further to this, during the development of our model, we assessed whether publication year was a significant variable and found it not predictive of IPF incidence or prevalence (either positively or negatively).

Our analysis also has limited geographic spread, with economically similar countries represented. In some countries, such as Germany, the healthcare system does not easily allow for structured data analysis [60]. In others, particularly low- or middle-income countries, few epidemiologic data are available, possibly due to reduced access to diagnostic tools and healthcare professionals with the expertise needed to provide an accurate diagnosis. Of the included studies, limited data were provided on covariates that could have been informative had they been available for analysis. For example, smoking status is a well-known risk factor associated with IPF prevalence [20, 21], but was not available for integration into our model. Other hard-to-quantify parameters, such as exposure to environmental hazards or overall healthcare system capacity, may also be influential features. For incidence, the development of a robust model was challenging, as data can be reported as a function of observed patient time (typically per patient-years) or as a function of the population observed. An adjustment was made to allow for the studies to be combined, and as such our results should be considered exploratory and in the context of the prevalence results. Finally, we note that the quality of data in the included studies may impact the validity of the study findings; however, due to the correlation between coding systems and diagnostic reliability, the impact is unlikely to be extensive [13, 59].

## Conclusions

Reported IPF incidence and prevalence are variable worldwide, even with statistical adjustment made where possible for between-study differences. Based on the countries included in our analysis, the adjusted incidence and prevalence of IPF are estimated to be in the range of 0.09–1.30 and 0.33–4.51 per 10,000 persons, respectively. According to these prevalence estimates, IPF remains a rare disease. Future epidemiologic studies of IPF should take age, sex, other risk factors, and the specificity of case definitions into consideration to better characterize the IPF patient population.

## Supplementary Information

**Additional file 1: Table S1.** PICO search criteria. **Table S2.** Model-associated adjustments for prevalence estimates adjusted per country. **Table S3.** Included studies and associated IPF categories. **Table S4.** List of studies for IPF incidence estimates. **Table S5.** List of studies for IPF prevalence estimates (primary analysis).

## Data Availability

All data generated or analysed during this study are included in this published article and its additional information files.

## Abbreviations

ALAT: Latin American Thoracic Society
ATS: American Thoracic Society
CI: Confidence interval
CT: Computed tomography
ERS: European Respiratory Society
FVC: Forced vital capacity
HRCT: High-resolution computed tomography
ICD: International Classification of Diseases
IIP: Idiopathic interstitial pneumonia
ILD: Interstitial lung disease
IPF: Idiopathic pulmonary fibrosis
JRS: Japanese Respiratory Society
NA: Not applicable
NHI: National Health Insurance
NHIS: National Health Insurance Service
NR: Not reported
PICO: Population, intervention, comparison, outcome
RID: Rare intractable diseases
UIP: Usual interstitial pneumonia
