# Supplementary material for: Global incidence and prevalence of idiopathic pulmonary fibrosis
Source: Respir Res. 2021 Jul 7;22:197. doi: 10.1186/s12931-021-01791-z (PMC8261998; doi:10.1186/s12931-021-01791-z)
Supplement: Supplementary file 1 — Additional file 1: Table S1. PICO search criteria. Table S2. Model-associated adjustments for prevalence estimates adjusted per country. Table S3. Included studies and associated IPF categories. Table S4. List of studies for IPF incidence estimates. Table S5. List of studies for IPF prevalence estimates (primary analysis). [file 12931_2021_1791_MOESM1_ESM.docx]

# ADDITIONAL FILE 1

**Table S1.** PICO search criteria.

|  | **Inclusion** | **Exclusion** |
| --- | --- | --- |
| Population | - Patients with IPF   - No restriction on case definitions | - Patients only with ILD or without specification on the proportion patients with IPF |
| Intervention | Any | – |
| Comparator | Any | – |
| Outcomes | Quantitative measures of:   - IPF prevalence (authors definition) - IPF incidence (authors definition) | - Non-quantitative measures of prevalence or incidence |
| Study design | - Population-based, observational study design | - Non-epidemiologic study design (randomized clinical trial, non-randomized clinical trial, animal study, experimental study, practice guideline, review article) - Publication type (case report, case series, commentary, editorial, author’s reply, letter, note, short survey, book chapter) |

*ILD* interstitial lung disease, *IPF* idiopathic pulmonary fibrosis, *PICO* population, intervention, comparison, outcome

**Table S2.** Model-associated adjustments for prevalence estimates adjusted per country.

| **Coefficient** | **Estimate** | **95% CI** | **P-value** |
| --- | --- | --- | --- |
| Intercept | 1.18×10^-6^ | (6.42×10^-8^, 2.16×10^-5^) | <0.0001 |
| Age | 1.06 | (1.01, 1.11) | 0.01 |
| Sex | 0.27 | (2.23×10^-3^, 29.3) | 0.58 |
| Canada | 8.93 | (4.29, 18.2) | <0.0001 |
| Denmark | 3.53 | (1.44, 8.66) | 0.01 |
| Finland | 1.96 | (0.93, 4.11) | 0.07 |
| France | 2.82 | (1.17, 6.81) | 0.02 |
| Italy | 7.13 | (3.47, 14.4) | <0.0001 |
| Japan | 2.68 | (1.30, 5.44) | 0.01 |
| Poland | 7.57 | (3.90, 14.70) | <0.0001 |
| South Korea | 13.54 | (7.13, 25.2) | <0.0001 |
| Taiwan | 1.71 | (0.86, 3.37) | 0.12 |
| United Kingdom | 2.35 | (1.00, 5.50) | 0.05 |
| United States | 7.22 | (3.37, 15.3) | <0.0001 |

*CI* confidence interval

**Table S3.** Included studies and associated IPF categories.

| **Author** | **Raw definition detail** | **Broad or specific** |
| --- | --- | --- |
| Agabiti N, et al. [1] | Annual prevalence and incidence of IPF were estimated at 25.6 per 100,000 and 7.5 per 100,000 using the ICD-9-CM code 516.3 without chart audit while they were estimated at 31.6 per 100,000 and at 9,3 per 100,000 for the IPF “confident” definition after hospital chart audit. | Both |
| Bartley K, et al. [2] | Single code: ICD-10 (J84.1) coded for patients who were aged ≥40 years and did not have a competing diagnosis after the initial J84.1 code; a diagnosis algorithm was used to refine the population for patients with IPF (*no further description in the abstract*). | Broad |
| Duchemann B, et al. [3] | Diagnoses (case confirmation) were validated centrally by an expert multidisciplinary discussion. Three clinicians two radiologists and one pathologist discussed each case, after a systematic review of medical charts, HRCT, and surgical lung biopsy, when applicable. The etiology of ILD was considered “determined” when a diagnosis could be attributed in consensus according to the current guidelines, including 2011 ATS/ERS/JRS/ALAT diagnostic criteria for IPF. The etiology of ILD remained “undetermined” when a diagnosis could not be established based on available investigations after multidisciplinary discussion. For unreviewable cases, information on the etiological diagnosis was taken from the completed questionnaires or the ICD-10 codes. | Specific |
| Esposito DB, et al. [4] | **Definition 1:** At least one claim for ICD-9 (516.3) by a physician + No alternative diagnoses recorded after the date of the last claim for 516.3 and within 6 months of the first physician-assigned diagnosis of IPF.  **Definition 2**: At least one claim for ICD-9 (516.3) by a physician + No alternative diagnoses recorded after the date of the last claim for 516.3 and within 6 months of the first physician-assigned diagnosis of IPF + At least one claim for ICD-9 (516.3) by a pulmonologist + At least one claim for ICD-9 (516.3) ≥4 days after HRCT of the chest or ≥4 weeks after open lung biopsy (for those patients with ≥12 months of continuous health plan eligibility before the first recorded IPF diagnosis) + Meet at least two criteria of the following: age ≥65 years, diagnosis of IPF persisting for ≥3 months, pulmonary function tests performed; hospitalization with IPF as the principal discharge diagnosis, IPF diagnosis recorded after antinuclear antibody or rheumatoid factor tests were performed or lung transplantation. | Broad |
| Fernández Pérez ER, et al. [5] | Patients who had an ICD-9-CM code 516.3 and the Hospital International Classification of Diseases–Adapted codes 517 and 519 were identified as potential cases. After potential cases were identified in the electronic database, IPF cases were identified by two methods: (1) evidence of UIP on surgical lung biopsy specimens or definite UIP pattern on HRCT images (specific case finding criteria) and (2) evidence of UIP on surgical lung biopsy specimens or a definite or possible UIP pattern on HRCT images (broad case finding criteria, representing the entire patient study cohort). Patients under the first method met all major and minor ATS/ERS criteria for diagnosis of IPF used in the absence of surgical lung biopsy. The second method included all those patients in the first method and a subgroup of patients that met the ATS/ERS criteria for diagnosis of IPF, but in whom the HRCT features were characterized as possible for UIP. | Both |
| Harari S, et al. [6] | **Broad case definition**: At least one hospital admission or outpatient visit with IPF diagnosis (ICD-9-CM code 516.3) and no hospital admission or outpatient visit with ILDs diagnosis (ICD-9-CM codes 135, 237.7, 272.7, 277.3, 277.8, 446.21, 446.4, 495, 500, 501, 502, 503, 504, 505, 506.4, 508.1, 508.8, 515, 516.0, 516.1, 516.2, 516.8, 516.9, 517.2, 517.8, 518.3, 555, 710.0, 710.1, 710.2, 710.3, 710.4, 714.81, 720.0, 759.5) on or after date of last IPF diagnosis (ICD-9-CM code 516.3).  **Specific case definition:** At least one hospital admission or outpatient visit with IPF diagnosis (ICD-9-CM code 516.3) and no hospital admission or outpatient visit with ILDs diagnosis (ICD-9-CM codes 135, 237.7, 272.7, 277.3, 277.8, 446.21, 446.4, 495, 500, 501, 502, 503, 504, 505, 506.4, 508.1, 508.8, 515, 516.0, 516.1, 516.2, 516.8, 516.9, 517.2, 517.8, 518.3, 555, 710.0, 710.1, 710.2, 710.3, 710.4, 714.81, 720.0, 759.5) on or after date of last IPF diagnosis (ICD-9-CM code 516.3) and at least one surgical lung biopsy (ICD-9-CM code 33.28), transbronchial lung biopsy (ICD-9-CM code 33.27), or CT of the thorax (ICD-9-CM codes 87.41) performed during an hospitalization or outpatient visit, on or before date of last IPF diagnosis (ICD-9-CM code 516.3). | Both |
| Hopkins RB, et al. [7] | **Broad case definition:** Excluding cases with an ICD-10-CA code for another ILD after the J84.1 code.  **Specific case definition:** Further excluded [broad] cases that did not have a code for chest CT, bronchus or lung biopsy, or bronchoscopy prior to establishing a J84.1 code. | Both |
| Hyldgaard C, et al. [8] | All available HRCT scans, patient histories, and pathological specimens used for disease evaluation were reevaluated according to the ATS/ERS Multidisciplinary International Consensus Classification of the IIPs and the 2011 ATS/ERS/JRS/ALAT criteria for IPF and other standard diagnostic criteria when available. Three radiologists and two pulmonologists specialized in the evaluation of ILDs were involved in the re-evaluation. | Narrow |
| Kaunisto J, et al. [9] | ICD-10 codes J84.1 (other interstitial pulmonary diseases with fibrosis) and J84.9 (interstitial pulmonary disease) were used to identify the potential cases. An experienced pulmonary physician evaluated patients’ medical records to assess diagnosis; 70–80% of patients’ diagnoses did not meet the clinical criteria of IPF (unknown exact number of screen failures). Surgical lung biopsy was performed in 27 (22%) patients. For the final total of 111 IPF patients, the mean FVC at diagnosis was 80.4%. | Specific |
| Kim SW, et al. [10] | At least two claims per year under the K-J84.18 code (IPF) of the Korean medical care system, using the KCD-6 codes. The K-J84.18 code classifies RID, and patients assigned this code can obtain up to 10% reduction in medical costs from the NHIS. The hospitals charge the NHIS for the remainder, and if their diagnosis does not meet to the RID diagnostic criteria, hospital costs are refused by the NHIS. Physicians are therefore cautious when making a diagnosis of RID in Korea. Care of patients assigned the K-J84.18 code is thus managed by the government, which provides high diagnostic validity. | Specific |
| Kondoh S, et al. [11] | Physicians who have made a diagnosis of IPF submit the clinical records, HRCT findings, and the result of pathologic diagnosis (when a surgical biopsy has been performed) to the local municipality. A committee comprising three pulmonologists specialized in ILD examines each case and grants certificates of medical benefit to those patients who satisfy the diagnostic criteria of IPF. | Specific |
| Lai CC, et al. [12] | **Broad case definition**: At least one claim with IPF diagnosis (ICD-9-CM code 516.3) + no claim with diagnosis for any other interstitial lung diseases since the day of IPF diagnosis (the time of confirming IPF incidence was the date of the first claim meeting this condition) + aged ≥18 years at the time of confirming IPF incidence.  **Specific case definition**: All conditions for the broad definition were satisfied + at least one NHI code for surgical lung biopsy, transbronchial lung biopsy, or CT of the thorax before or on the day of IPF diagnosis (the time of confirming IPF incidence was “the date of the first claim meeting this condition,” which might be later than the time for IPF incidence under the broad definition). | Both |
| Lee HE, et al. [13] | Patients with IPF were defined as those with a disease code of K-J84.18 (other interstitial pulmonary diseases with fibrosis). For certain rare diseases, including IPF (K-J84.18), the NHI operates a RID registration program. Beginning on 1 September 2009, all patients with RIDs are offered reductions in medical expenses when their diagnoses are certified by physicians based on designated criteria. Claims with diagnoses of RIDs are investigated by the NHI and then integrated into the Health Insurance and Review Agency database. A person newly diagnosed with IIP in a calendar year was defined as an incident case. As previous prevalence may disturb incidence, the clearance period was set at 2 years. Incident cases thus included only patients newly diagnosed in calendar years 2011 and 2012. Data from 2013 were excluded, as some of these patients visited the outpatient service for IIP just once, a criterion for exclusion, but their medical utilization during the next year could not be followed up. | Specific |
| Natsuizaka M, et al. [14] | Patients with certificate of medical benefit for IPF between 2003 and 2007. | Specific |
| Raghu G, et al. [15, 16] | **Broad case definition**: Excluded those with a claim for ICD-9 (515) on or after the quarter of the last claim for ICD-9-CM (516.3).  **Specific case definition:** Further exclude broad cases with no claim for a surgical lung biopsy, transbronchial lung biopsy, or CT scan of the thorax before the last 516.3 (idiopathic fibrosing alveolitis). | Both |
| Raimundo K, et al. [17] | At least one inpatient claim or two outpatient claims anytime in that calendar year with IPF coded as ICD-9-CM (516.3). Patients were required to be continuously enrolled with the health plan in the same calendar year and have no claims for other type of ILD diagnosis after their last IPF claim in that calendar year. In 2012, the ICD-9 code for IPF changed from 516.3 to 516.31. Due to the possibility of coding errors in 2012 resulting from this change, the study was limited to 2009–2011. | Broad |
| Snell N, et al. [18] | Using a broad range of primary care codes considered to encompass the IPF definition (*codes were not provided in the abstract*). | Broad |
| Strongman H, et al. [19] | **Broad case definition**: Identified patients with Read codes: H563.00 (Idiopathic fibrosing alveolitis), H563.12 (Cryptogenic fibrosing alveolitis), H563z00 (Idiopathic fibrosing alveolitis NOS), H563300 (Usual interstitial pneumonitis), H563.13 (Idiopathic pulmonary fibrosis), H563100 (Diffuse pulmonary fibrosis), H563200 (Pulmonary fibrosis), and H563.11 (Hamman–Rich syndrome). Patients with Read codes for connective tissue disease, extrinsic allergic alveolitis, sarcoidosis, pneumoconiosis, or asbestosis at any time in their medical records were not included as cases.  **Specific case definition:** Identified patients with Read codes H563.00 (Idiopathic fibrosing alveolitis), H563.12 (Cryptogenic fibrosing alveolitis), H563z00 (Idiopathic fibrosing alveolitis NOS), H563300 (Usual interstitial pneumonitis), and H563.13 (Idiopathic pulmonary fibrosis). Patients with Read codes for connective tissue disease, extrinsic allergic alveolitis, sarcoidosis, pneumoconiosis, or asbestosis at any time in their medical records were not included as cases. | Both |
| Szafrański W [20] | Diagnosis of ILD was based on clinical data, laboratory examination, HRCT, lung function tests, and in some patients on the results of open or transbronchial lung biopsies. | Specific |
| Tarride JE, et al. [21] | **Broad case definition**: Had either the ICD-9 or ICD-10 code of IPF for hospital admissions or physician billings databases over the 5 years without having a subsequent code for >200 other interstitial diseases (including connective tissue diseases, sclerosis, sarcoidosis, etc).  **Specific case definition:** Satisfied the broad case definition + the first IPF code diagnosis recorded after a CT scan of the thorax, surgical lung biopsy, or bronchoscopy. | Both |

*ALAT* Latin American Thoracic Society, *ATS* American Thoracic Society, *CT* computed tomography, *ERS* European Respiratory Society, *FVC* forced vital capacity, *HRCT* high-resolution computed tomography, *ICD* International Classification of Diseases, *IIP* idiopathic interstitial pneumonia, *ILD* interstitial lung disease, *IPF* idiopathic pulmonary fibrosis, *JRS* Japanese Respiratory Society, *NHI* National Health Insurance, *NHIS* National Health Insurance Service, *RID* rare intractable disease, *UIP* usual interstitial pneumonia

**Table S4.** List of studies for IPF incidence estimates.

| **Author** | **Published year** | **Study region** | **Study country** | **Males, %** | **Age, years** | **Total study period, years** | **Raw reported cases** | **Reported incidence (per 10,000)** | **Count or patient-year** | **Reports patient-year observed?** | **Data source** |
| --- | --- | --- | --- | --- | --- | --- | --- | --- | --- | --- | --- |
| Han S, et al. [22] | 2013 | Asia-Pacific | South Korea | 63.9 | 45.3 | 15 | 24 | 0–0.78 | Person-Year | No | Secondary (claim) |
| Kim SW, et al. [10] | 2017 | Asia-Pacific | South Korea | 61.9 | 67.5 | 5 | 6556 | 1.31 | Count | NA | Secondary (claim) |
| Lai CC, et al. [12] | 2012 | Asia-Pacific | Taiwan | 61.9 | 66.8 | 11 | 82 | 0.01–0.21 | Count | NA | Secondary (claim) |
| Lee HE, et al. [13] | 2016 | Asia-Pacific | South Korea | 74 | 67.5 | 5 | 5950 | 0.97 | Count | NA | Secondary (claim) |
| Agabiti N, et al. [1] | 2014 | Europe | Italy | 53 | 70.3 | 5 | 1752 | 0.93 | Count | NA | Primary (medical chart) |
| Duchemann B, et al. [3] | 2017 | Europe | France | 76.5 | 70.3 | 1 | 33 | 0.28 | Person-Year | Yes | Primary (medical chart) |
| Harari S, et al. [6] | 2016 | Europe | Italy | 57 | 68.4 | 11 | 1309 | 0.26 | Person-Year | No | Secondary (claim) |
| Karakatsani A, et al. [23] | 2009 | Europe | Greece | NR | NR | 1 | 52 | 0.093 | Count | NA | Primary (medical chart) |
| Kaunisto J, et al. [9] | 2015 | Europe | Finland | 60.4 | 73.5 | 3 | 17 | NR | Count | NA | Primary (medical chart) |
| Strongman H, et al. [19] | 2018 | Europe | United Kingdom | 56 | 74.6 | 13 | 93 | 0.285 | Person-year | Yes | Secondary (claim) |
| Fernández Pérez ER, et al. [5] | 2010 | North America | United States | 59 | 73.5 | 9 | 24 | 0.88 (age and sex adjusted) | Person-year | No | Primary (medical chart) |
| Hopkins RB, et al. [7] | 2016 | North America | Canada | 59.3 | 74.4 | 5 | 3057 | 0.9 | Count | NA | Secondary (claim) |
| Ley B, et al. [24] | 2017 | North America | United States | 49 | 74.6 | 15 | 186 | 0.68 | Person-year | No | Secondary (claim) |
| Raghu G, et al. [15, 16] | 2014/2016 | North America | United States | 47.1 | 55.3 | 12/7 | 3195 (65+) 1685 (<65) | 1.5–3.1 (65+) 0.24–0.29 (<65) | Person-year | No | Secondary (claim) |
| Tarride JE, et al. [21] | 2018 | North America | Canada | 60 | 76.8 | 5 | 5152 | 2.17 | Count | NA | Secondary (claim) |

*IPF* idiopathic pulmonary fibrosis, *NA* not available, *NR* not reported

**Table S5.** List of studies for IPF prevalence estimates (primary analysis).

| **Author** | **Published year** | **Study region** | **Study country** | **Males, %** | **Age, years** | **Reported prevalence**  **(per 10,000)** | **Data source** |
| --- | --- | --- | --- | --- | --- | --- | --- |
| Kim SW, et al. [10] | 2017 | Asia-Pacific | South Korea | 61.9 | 67.5 | 3.52 | Secondary (claim) |
| Kondoh S, et al. [11] | 2016 | Asia-Pacific | Japan | 74.8 | 69.7 | 0.59 | Primary (medical chart) |
| Lai CC, et al. [12] | 2012 | Asia-Pacific | Taiwan | 61.9 | 66.8 | 0.49 | Secondary (claim) |
| Lee HE, et al. [13] | 2016 | Asia-Pacific | South Korea | 74 | 67.5 | 3.89 | Secondary (claim) |
| Natsuizaka M, et al. [14] | 2014 | Asia-Pacific | Japan | 72.7 | 70 | 0.99 | Primary (medical chart) |
| Agabiti N, et al. [1] | 2014 | Europe | Italy | 53 | 70.3 | 3.16 | Primary (medical chart) |
| Duchemann B, et al. [3] | 2017 | Europe | France | 76.5 | 70.3 | 0.82 | Primary (medical chart) |
| Harari S, et al. [6] | 2016 | Europe | Italy | 57 | 68.4 | 2.12 | Secondary (claim) |
| Hyldgaard C, et al. [8] | 2013 | Europe | Denmark | 77 | NR | 1.01 | Primary (medical chart) |
| Karakatsani A, et al. [23] | 2009 | Europe | Greece | NA | NR | 0.34 | Primary (medical chart) |
| Kaunisto J, et al. [9] | 2015 | Europe | Finland | 60.4 | 73.5 | 0.86 | Secondary (claim) |
| Strongman H, et al. [19] | 2018 | Europe | United Kingdom | 56 | 74.6 | 1.16 | Secondary (claim) |
| Szafrański W [20] | 2012 | Europe | Poland | NR | NR | 2.56 | Primary (medical chart) |
| Fernández Pérez ER, et al. [5] | 2010 | North America | United States | 59 | 73.5 | 2.81 | Primary (medical chart) |
| Hopkins RB, et al. [7] | 2016 | North America | Canada | 59.3 | 74.4 | 2.0 | Secondary (claim) |
| Raghu G, et al. [15, 16] | 2014/2016 | North America | United States | 47.1 | 55.3 | 1.37 | Secondary (claim) |
| Tarride JE, et al. [21] | 2018 | North America | Canada | 60 | 76.8 | 7.27 | Secondary (claim) |

*IPF* idiopathic pulmonary fibrosis, *NA* not available, *NR* not reported

**REFERENCES**

1. Agabiti N, Porretta MA, Bauleo L, Coppola A, Sergiacomi G, Fusco A, et al. Idiopathic pulmonary fibrosis (IPF) incidence and prevalence in Italy. Sarcoidosis Vasc Diffuse Lung Dis. 2014;31:191−7.

2. Bartley K, Levine A, Arnheim-Dahlstrom L, Ferrara G, Kirchgaessler K, Linder R, et al. Description of a national pulmonary fibrosis cohort in Sweden. Thorax. 2017;72(Suppl 3):A164–5 [Abstract P49].

3. Duchemann B, Annesi-Maesano I, Jacobe de Naurois C, Sanyal S, Brillet PY, Brauner M, et al. Prevalence and incidence of interstitial lung diseases in a multi-ethnic county of Greater Paris. Eur Respir J. 2017;50:1602419.

4. Esposito DB, Lanes S, Donneyong M, Holick CN, Lasky JA, Lederer D, et al. Idiopathic pulmonary fibrosis in United States automated claims. Incidence, prevalence, and algorithm validation. Am J Respir Crit Care Med. 2015;192:1200−7.

5. Fernández Pérez ER, Daniels CE, Schroeder DR, St Sauver J, Hartman TE, Bartholmai BJ, et al. Incidence, prevalence, and clinical course of idiopathic pulmonary fibrosis: a population-based study. Chest. 2010;137:129−37.

6. Harari S, Madotto F, Caminati A, Conti S, Cesana G. Epidemiology of idiopathic pulmonary fibrosis in northern Italy. PLoS One. 2016;11:e0147072.

7. Hopkins RB, Burke N, Fell C, Dion G, Kolb M. Epidemiology and survival of idiopathic pulmonary fibrosis from national data in Canada. Eur Respir J. 2016;48:187−95.

8. Hyldgaard C, Hilberg O, Muller A, Bendstrup E. A cohort study of interstitial lung diseases in central Denmark. Respir Med. 2014;108:793−9.

9. Kaunisto J, Kelloniemi K, Sutinen E, Hodgson U, Piilonen A, Kaarteenaho R, et al. Re-evaluation of diagnostic parameters is crucial for obtaining accurate data on idiopathic pulmonary fibrosis. BMC Pulm Med. 2015;15:92.

10. Kim SW, Myong JP, Yoon HK, Koo JW, Kwon SS, Kim YH. Health care burden and medical resource utilisation of idiopathic pulmonary fibrosis in Korea. Int J Tuberc Lung Dis. 2017;21:230−5.

11. Kondoh S, Chiba H, Nishikiori H, Umeda Y, Kuronuma K, Otsuka M, et al. Validation of the Japanese disease severity classification and the GAP model in Japanese patients with idiopathic pulmonary fibrosis. Respir Investig. 2016;54:327−33.

12. Lai CC, Wang CY, Lu HM, Chen L, Teng NC, Yan YH, et al. Idiopathic pulmonary fibrosis in Taiwan - a population-based study. Respir Med. 2012;106:1566−74.

13. Lee HE, Myong JP, Kim HR, Rhee CK, Yoon HK, Koo JW. Incidence and prevalence of idiopathic interstitial pneumonia and idiopathic pulmonary fibrosis in Korea. Int J Tuberc Lung Dis. 2016;20:978−84.

14. Natsuizaka M, Chiba H, Kuronuma K, Otsuka M, Kudo K, Mori M, et al. Epidemiologic survey of Japanese patients with idiopathic pulmonary fibrosis and investigation of ethnic differences. Am J Respir Crit Care Med. 2014;190:773−9.

15. Raghu G, Chen SY, Yeh WS, Maroni B, Li Q, Lee YC, et al. Idiopathic pulmonary fibrosis in US Medicare beneficiaries aged 65 years and older: incidence, prevalence, and survival, 2001-11. Lancet Respir Med. 2014;2:566−72.

16. Raghu G, Chen SY, Hou Q, Yeh WS, Collard HR. Incidence and prevalence of idiopathic pulmonary fibrosis in US adults 18-64 years old. Eur Respir J. 2016;48:179−86.

17. Raimundo K, Chang E, Broder MS, Alexander K, Zazzali J, Swigris JJ. Clinical and economic burden of idiopathic pulmonary fibrosis: a retrospective cohort study. BMC Pulm Med. 2016;16:2.

18. Snell N, Strachan D, Hubbard R, Gibson J, Maher T, Jarrold I. Epidemiology of idiopathic pulmonary fibrosis in the UK: findings from the British Lung Foundation’s ‘Respiratory Health of the Nation’ project [Abstract P272]. Thorax. 2016;71(Suppl 3):A236.

19. Strongman H, Kausar I, Maher TM. Incidence, prevalence, and survival of patients with idiopathic pulmonary fibrosis in the UK. Adv Ther. 2018;35:724−36.

20. Szafrański W. Interstitial lung diseases among patients hospitalized in the department of respiratory medicine in radom district hospital during the years 2000-2009. Pneumonol Alergol Pol. 2012;80:523−32.

21. Tarride JE, Hopkins RB, Burke N, Guertin JR, O'Reilly D, Fell CD, et al. Clinical and economic burden of idiopathic pulmonary fibrosis in Quebec, Canada. Clinicoecon Outcomes Res. 2018;10:127−37.

22. Han SH, Mok YJ, Jee SH, Danoff SK. Incidence and mortality of idiopathic pulmonary fibrosis in South Korea. Am J Respir Crit Care Med. 2013;201:A1460.

23. Karakatsani A, Papakosta D, Rapti A, Antoniou KM, Dimadi M, Markopoulou A, et al. Epidemiology of interstitial lung diseases in Greece. Respir Med. 2009;103:1122−9.

24. Ley B, Urbania T, Husson G, Vittinghoff E, Brush DR, Eisner MD, et al. Code-based diagnostic algorithms for idiopathic pulmonary fibrosis. Case validation and improvement. Ann Am Thorac Soc. 2017;14:880−7.
